# Rehabilitation Research in Denmark Between 2001 and 2020: A Scoping Review

**DOI:** 10.3389/fresc.2022.849216

**Published:** 2022-03-10

**Authors:** Anne-Mette Hedeager Momsen, Jasmine Charlotte Fox, Claus Vinther Nielsen, Jette Thuesen, Thomas Maribo

**Affiliations:** ^1^DEFACTUM-Social and Health Services and Labour Market, Corporate Quality, Central Denmark Region, Aarhus, Denmark; ^2^Department of Public Health, Aarhus University, Aarhus, Denmark; ^3^Department of Clinical Social Medicine and Rehabilitation, Gødstrup Hospital, Herning, Denmark; ^4^Knowledge Centre for Rehabilitation and Palliative Care (REHPA), Odense University Hospital, Odense, Denmark; ^5^Department of Public Health, University of Southern Denmark, Odense, Denmark; ^6^Centre for Nutrition and Rehabilitation, Absalon University College, Sorø, Denmark

**Keywords:** rehabilitation, Denmark, research institutions, scoping review, health condition, rehabilitation research

## Abstract

The demand for rehabilitation has increased, and evidence is rapidly growing; however, a rehabilitative health strategy receives less attention than treatment. Knowledge of what is being researched, who are the target groups and who contributes to rehabilitation research is deficient. We did not find any reviews mapping rehabilitation research regarding the research questions. The objective was to identify and synthesize existing scientific evidence on rehabilitation research published by Danish institutions between 2001 and 2021. The research questions to be explored were: Among which study groups has rehabilitation research been published?. Which types of studies on rehabilitation have been published?. Which institutions have been involved in rehabilitation research?

**Methods:**

The process was guided according to the Joanna Briggs Institute's (JBI's) scoping review methodology. Four databases were searched. All types of peer-reviewed studies on any target group and rehabilitation setting, with any affiliation to a Danish institution, were eligible to be included. Studies referring to population and the type of design were categorized. Institutions were counted as Danish first authorship.

**Results:**

The search revealed 3,100 studies, and following screening 1,779 were included. A total of 24 broad study groups were identified, mostly diagnosis-based health conditions. Musculoskeletal, cancer, and cardiac had 342, 228, and 174 studies, respectively. A total of 1,545 had a Danish first authorship, most of the Danish publications came from hospitals (56.6%) and universities (28.4%). The publication trend showed an almost linear development, with a 10–15% increase during the period.

**Conclusion:**

Following screening 1,779 studies were included involving 24 broad study groups. Most categories were diagnosis-based; musculoskeletal, cancer, and cardiac health conditions encompassed most studies. All study designs were represented, and 1/10 were secondary studies. The majority (87%) of studies had a Danish first authorship. The majority of first affiliations were among hospitals followed by universities. A few municipalities were presented although they are yet to have research responsibility. Publication trends showed an increase primarily from 2013.

**Systematic Review Registration:**

https://osf.io/, identifier [10.17605/OSF.IO/2AENX].

## Introduction

Rehabilitation has been identified by the WHO as an essential health strategy alongside promotion, prevention, treatment, and palliative care (1). However, internationally, the rehabilitative health strategy has received less attention among health policymakers (2) although the need for rehabilitation measures is increasing. Recently, it has been estimated that more than 2 billion people worldwide are in need of rehabilitation (3).

The term rehabilitation is heterogeneously used in health contexts, as well as in education, law, and engineering (4). Functioning can be considered as the lived experience of health (5).

Since the launch of WHO's International Classification of Functioning, Disability, and Health (ICF) in 2001 (6), other definitions have been introduced, e.g., rehabilitation as a process, as a set of interventions, and as a health strategy (4, 7). Thus, there is no international consensus on rehabilitation or the constituent elements (4, 8). This may be the reason why knowledge of what is being researched, who the target groups are and who contributes to rehabilitation research is scarce.

A preliminary search on Epistemonikos and PubMed identified scoping reviews regarding rehabilitation including specific study groups or services, e.g., vocational rehabilitation (9), obese people (10), elderly people (11), assessing instruments (12), and the use of artificial intelligence (13). Furthermore, a scoping review analyzing rehabilitation scoping reviews concluded that the number of reviews is increasing, but some are of suboptimal methodological quality (14). We did not find reviews mapping rehabilitation research. Thus, this scoping review investigates which types of rehabilitation studies were performed, which study groups were analyzed, and analysis by which institutions.

In accordance with global trends, the Danish population is aged, and the prevalence of chronic diseases and multimorbidity is increasing (15). Furthermore, the recognition of social inequality in health is increasing (16), leading to the expansion of target groups for rehabilitation. Thus, subgroups exist where not only disease, but also complex contextual factors uncover an obvious need for rehabilitation. Politically and administratively, rehabilitation as a concept has been integrated in Danish social, aging, and employment policy and legislation (17–19).

Further, rehabilitation as a field and a professional discipline has developed and has been integrated in formal education, and different knowledge perspectives have been developed. A Danish “white paper” was published in 2004 (20), and a new one is underway. The phenomena are internationally relevant, not in Denmark (DK) only, but the objective of this scoping review was limited to identifying and synthesizing existing rehabilitation research published by Danish institutions (practitioners and researchers) between 2001 and 2021. The aim was a mapping of the available literature on the range of any type of rehabilitation research provided among any groups.

The research questions were:

Among which study groups has rehabilitation research been published?

Which types of studies on rehabilitation research have been published?

Which Danish institutions have been involved in rehabilitation research?

## Method

The process was guided according to the Joanna Briggs Institute's (JBI's) model (21, 22). Furthermore, the Preferred Reporting Items for Systematic Reviews and Meta-Analyses extension for Scoping Reviews (PRISMA-ScR) reporting guideline and checklist were included (23). *A priori* protocol was registered in the OSF: DOI 10.17605/OSF.IO/2AENX.

### Inclusion Criteria

Peer-reviewed primary and secondary studies on rehabilitation in English or Danish with an affiliation to DK were eligible. All study populations were eligible. The context was open and encompassed all rehabilitation approaches and settings, including international cooperation on rehabilitation guidelines (involving Danish institutions).

### Search Strategy

Initially, the Cochrane Database of Systematic Reviews, PubMed, and Epistemonikos were searched for other scoping reviews on the topic rehabilitation. We identified relevant studies by searching: PubMed (PubMed), Embase (OvidSP), PsychINFO (ProQuest), and Cumulative Index to Nursing and Allied Health Literature (CINAHL) (EBSCOhost) for peer-reviewed articles. The search period was from the introduction of the ICF January 1, 2001 to March 1, 2021.

The design and refining of the search and use of Covidence© for data mapping were qualified by the input of a research librarian having experience in the topic. The complete search strategy is available in Appendix 1.

### Study Selection

The search results were transferred into the platform, Covidence©. Two authors (AM and JF) independently screened titles and abstracts (Level 1); disagreements were solved by a consensus.

All primary studies (quantitative and qualitative) and secondary studies (reviews, systematic reviews, meta-analyses, and meta-syntheses) were eligible. Papers examining rehabilitation were identified without restrictions on the type of design, intervention, phenomenon of interest, context/setting, and outcomes. Thus, the sources of information were open regarding concept and context to allow for any types of peer-reviewed and published evidence.

The term “Rehab” should be included in the title or abstract to make a study eligible. Furthermore, the inclusion criteria stated that at least one author should be affiliated in a Danish context (research, education, or rehabilitation setting geographically located in DK).

Exclusion criteria included study protocols, non-Danish authorship, non-Danish study population, conference abstracts, book chapters, thesis, letters, and websites. The second screening (Level 2) was performed by two independent reviewers (AM and JF); disagreement was resolved by the third reviewer (TM). The selection process was recorded in a PRISMA flow diagram (24).

### Data Extraction

Based on 10 included systematic reviews, a guide for data extraction was developed. Data were extracted by one reviewer, and accuracy was checked by a second reviewer.

### Analysis

The included studies were categorized with two separate tags referring to population and the type of design. The categories included broad groups of health conditions, e.g., brain injury, cancer, cardiac, functional disorder, lungs/pulmonary disease, musculoskeletal disease (MSD), neurological disease, oral health, stroke, and mental and cognitive impairment.

Studies were included in the elderly category if the title stated elderly participants despite their health condition. Studies were included in mixed population if the title or abstract stated that participants had two diseases or more. In the first round, the category “other” included diseases, such as autoimmune and rare diseases, surgery, caregivers, and healthy test people. In the second round, the “other” studies were subdivided before an analysis into new groups: biomechanical aspects, diabetes, hearing impairment, intensive care, refugees/torture, rheumatic, sport, substance abuse, and surgery. The cardiac group was further categorized into cardiac and stroke. Furthermore, two groups were categorized as people on sickness absence in vocational rehabilitation and healthcare professionals (HCPs), respectively. HCPs were the only group representing professionals, whereas a few studies, including social workers, were categorized according to the health condition among the rehabilitees.

Categories of study design were inspired by the Cochrane Handbook (quantitative/qualitative secondary and primary studies) (25). The studies were sorted into primary and secondary study types and then subdivided. Four secondary study types were identified: review, a systematic review (including qualitative and quantitative data), meta-analysis, and meta-synthesis. Nine primary study types were identified: RCT, non-RCT (quasi-), cohort (including longitudinal, prospective, retro-, follow-up, cost-effectiveness, and pretest-posttest), cross-sectional, survey (prospective and longitudinal questionnaire), qualitative (including mixed methods), case studies, and guidelines (policy papers, recommendations, and discussion). Before analysis, the design type validation (cohort studies/RCTs regarding instruments) was added as a category.

All studies' affiliations were recorded in an excel spreadsheet in relation to the category of the first author (Danish/non-Danish), the type of institution (hospital, municipal, regional, etc.), and Danish region (Capital Region of DK, Central DK Region, Region of Southern DK, Region Zealand, and North DK Region).

Studies were included in the count of institutions involved based on the primary affiliation for the first author.

## Results

**Figure 1:**
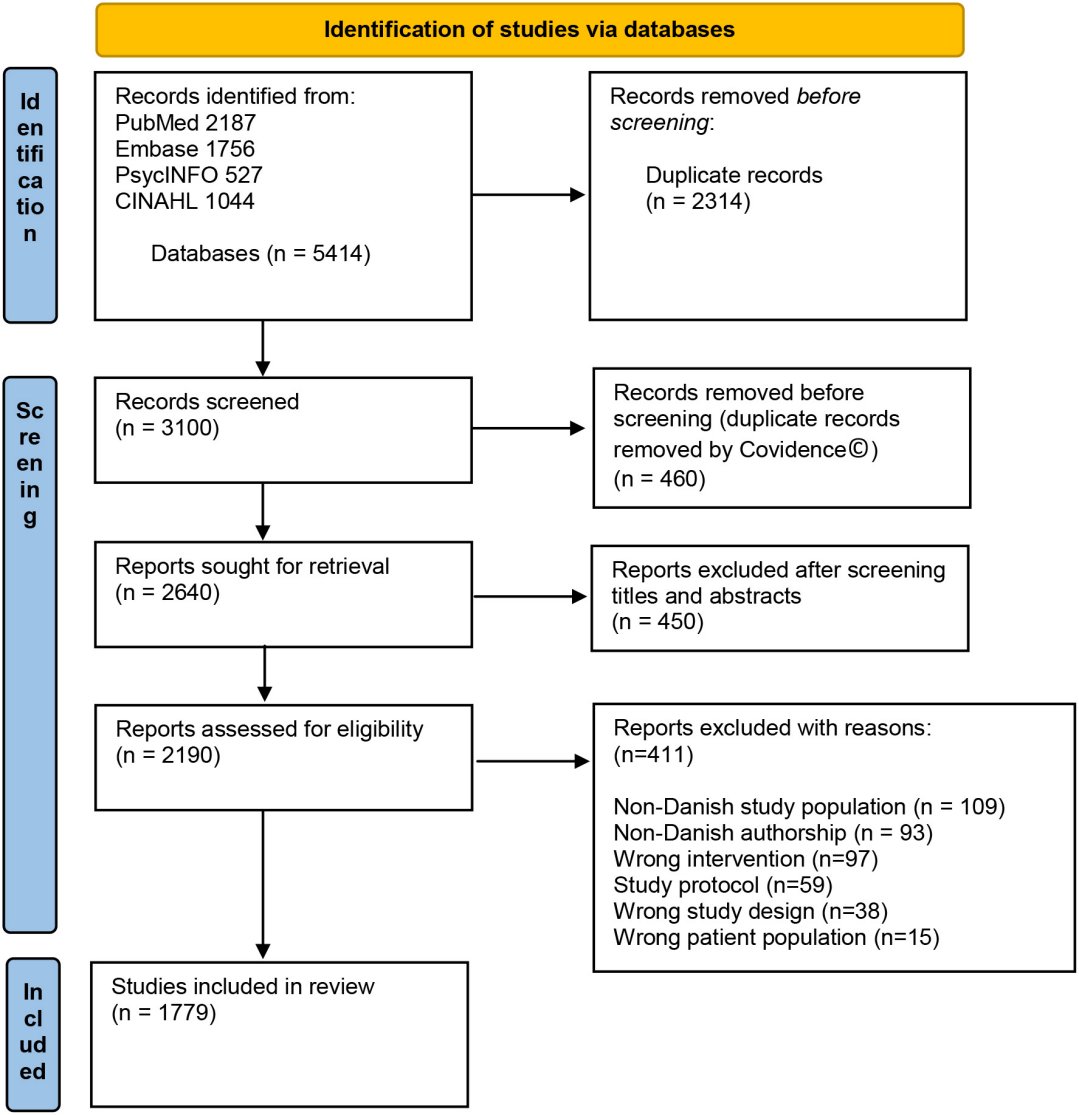
Flow diagram of studies. Modified from Page et al. (24).

As presented in Figure 1, the database searches resulted in 2,640 studies after duplicates were removed. Following Level 1 screening, 2,190 studies were assessed for eligibility in Level 2, and 411 of these were excluded based on inclusion criteria.

Thus, 1,779 studies were found eligible to be included for the final data extraction. The summation of rehabilitation research among study groups and study designs is provided in Table 1.

**Table 1:** Study groups and designs.

**Study group**							**Study design**							
		**Secondary studies**							**Primary studies**				
	**Review**	**Systematic review**	**Meta analysis**	**Meta synthesis**	**RCT**	**Non-RCT**	**Cohort**	**Cross sectional**	**Survey**	**Validation**	**Qualitative**	**Case**	**Guideline**	**Total**
Biomechanical	0	1	0	0	3	0	3	0	0	1	0	0	19	27
Brain injury	2	7	1	0	4	6	42	4	1	9	11	4	20	111
Cancer	12	8	1	1	29	5	56	10	10	2	70	4	20	228
Cardiac	5	6	12	0	34	5	41	9	12	3	30	0	17	174
Diabetes	0	1	1	0	3	0	0	1	0	0	1	0	1	7
Elderly	7	6	1	2	25	5	26	5	2	6	9	2	13	108
Functional disorder	0	0	0	0	4	2	0	1	0	2	2	0	3	14
Hearing impairment	1	2	0	0	1	1	8	1	0	1	9	3	6	33
Intensive care	0	1	0	0	1	0	2	0	0	0	5	0	1	10
Lungs/pulmonary	3	2	3	0	13	3	22	7	2	3	19	2	14	93
Mental/Cognitive	2	4	0	2	4	2	5	1	1	0	7	2	3	33
Mixed population	0	3	0	0	1	1	4	1	0	0	5	0	2	17
Musculoskeletal	12	14	5	1	75	17	71	20	2	16	24	8	77	342
Neurological	6	7	3	1	15	10	25	13	2	16	13	12	27	151
Oral health	3	1	0	0	0	4	7	0	0	1	2	4	7	29
Rheumatic	0	0	0	0	0	0	0	0	1	0	3	0	1	5
Sickness absence	0	2	0	0	6	1	18	2	2	3	14	0	4	52
Stroke	3	6	1	1	11	3	27	2	3	2	19	2	17	97
Substance abuse	0	0	0	0	0	0	2	2	0	0	4	0	0	8
Surgery	8	0	0	0	5	7	14	0	0	0	2	0	7	43
Refugees/torture	0	0	0	0	0	0	8	3	0	0	6	3	3	23
Sport	3	0	0	0	0	1	0	0	0	4	0	1	5	15
Other	4	2	2	0	6	4	24	7	6	11	11	5	39	121
Healthcare professional	1	1	0	0	0	2	1	1	7	0	19	0	6	38
Total	72	74	30	8	240	79	406	90	51	80	285	52	312	1779

### Study Groups

After screening, a total of 24 broad study groups were identified (Table 1). The majority of study groups were health conditions, such as brain injury, cancer, cardiac; whereas somewere based on other criteria: elderly, substance abuse, refugees/torture, sport, sickness absentees, and HCPs. The characteristics of participants in study groups are provided in Table 2.

**Table 2:** Characteristics of study groups.

**Study group**	**Studies' included health conditions and disorders in all age groups**	**No. of studies**
Biomechanical aspects	Studies that apply mechanical research to living structures such as the skeleton or organs. Assistive robotic devices e.g., exoskeletons, brain-computer interfaces (BCIs), functional electrical therapy (FET)	27
Brain injury	All types of head trauma resulting in acquired brain injury	111
Cancer	All types of cancer	228
Cardiac	Cardiac diseases such as coronary heart disease, heart transplant, venous thromboembolism, angina pectoris, heart valve replacement, and defibrillator rehabilitation	174
Diabetes	Diabetic foot ulcers, type 2 diabetes, socioeconomic inequality	7
Elderly	Elderly participants with any disease or condition mentioned in the title e.g. hip fractures, cognitive impairments and dementia	109
Functional disorder	Fibromyalgia, chronic fatigue, psychosomatic symptoms	14
Hearing impairment	Hearing loss, auditory verbal skills, cochlear implant	33
Intensive care	All types of ICU care and follow up	10
Lungs/pulmonary	All types of respiratory diseases, COPD, tuberculosis and asthma	93
Mental/Cognitive	All types of mental disorder, dementia and psychiatric diagnosis	33
Mixed population	Participants with more than one disease group mentioned in the title	17
Musculoskeletal	Low back pain, amputation, atrophy, hip knee replacement, fractures, tendon/ligament damage, muscle pain, osteoarthritis	342
Neurological	Cerebral paresis, multiple sclerosis, Parkinson's disorder, spinal cord injury and spasticity	151
Oral Health	Dental procedures, preventive measures	29
Rheumatic	Rheumatic disorders,	4
Sickness absence	Included studies with work related interventions and outcomes, e.g. return to work, and sick leave. Participants without a specific disease	52
Stroke	All types of stroke	97
Substance abuse	Drug treatment, alcoholic liver disease, cannabis withdrawal, hepatic encephalopathy	8
Surgery	Colon surgery, nephrectomy, postoperative recovery	43
Refugees/torture	Rehabilitating torture survivors, traumatised refugees	23
Sport	Sports injuries	15
Other	Included studies that did not conform to the previously mentioned groups e.g. genetic disorders, rare diseases	121
Healthcare professional	Nurses, occupational therapists, physiotherapists, and general practitioners	38

**Figure 2:**
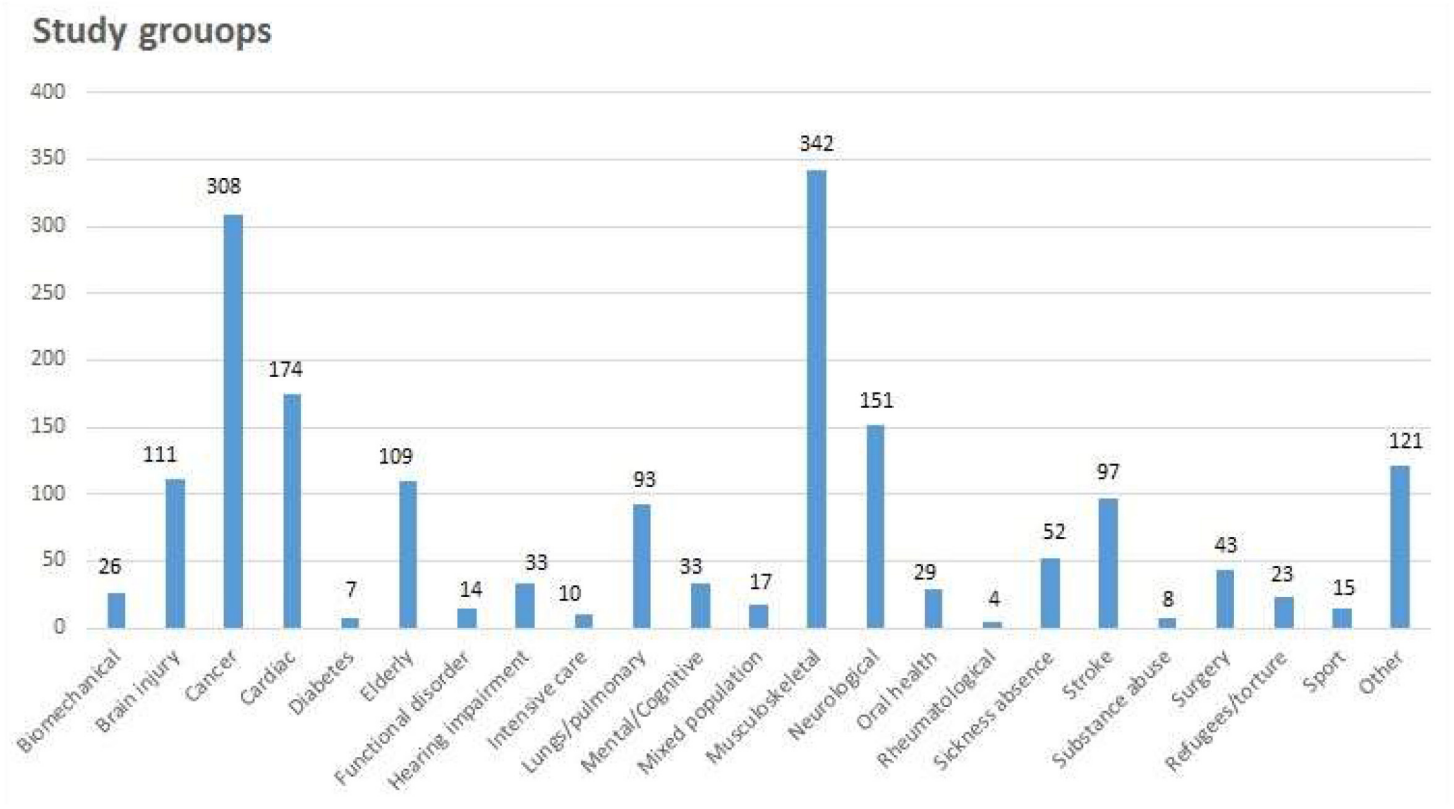
Study groups.

The largest groups were MSD, cancer, and cardiac with 342, 228, and 174 studies, respectively. Cardiac and stroke represented 15.2% of the total number of studies, Figure 2 shows the proportion of study groups included. MSD included a number of common health conditions, e.g., low back pain and hip fractures. Rheumatic diseases, diabetes, and substance abuse had only 5, 7, and 8 studies, respectively.

### Study Designs

**Figure 3:**
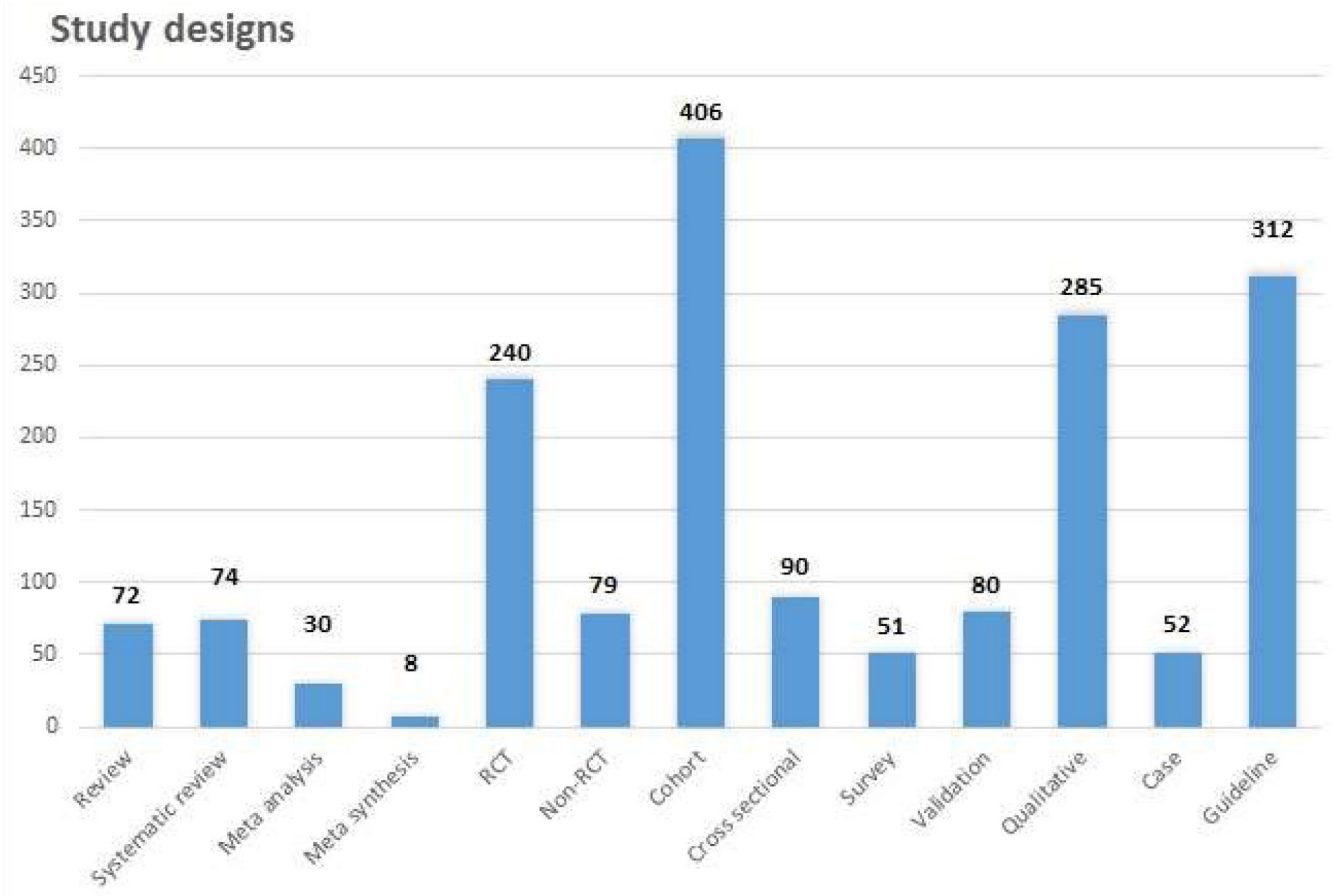
Study designs.

The secondary and primary studies were further categorized; Figure 3 shows the proportion of types of design. Only 10% of all study types were secondary studies, most prevalent of these were systematic reviews and reviews, which equated to 4% each.

A total of 34 secondary studies were published in cardiac and stroke, whereas biomechanical aspects, diabetes, and intensive care only had 1 secondary study each. The groups, such as rheumatic diseases, functional disorder, refugees/torture, and substance abuse, did not have any secondary studies. There were only qualitative meta-syntheses published in cancer, elderly, mental/cognitive, MSD, neurology, and stroke study groups.

### Institutions Involved in Rehabilitation Research

**Figure 4:**
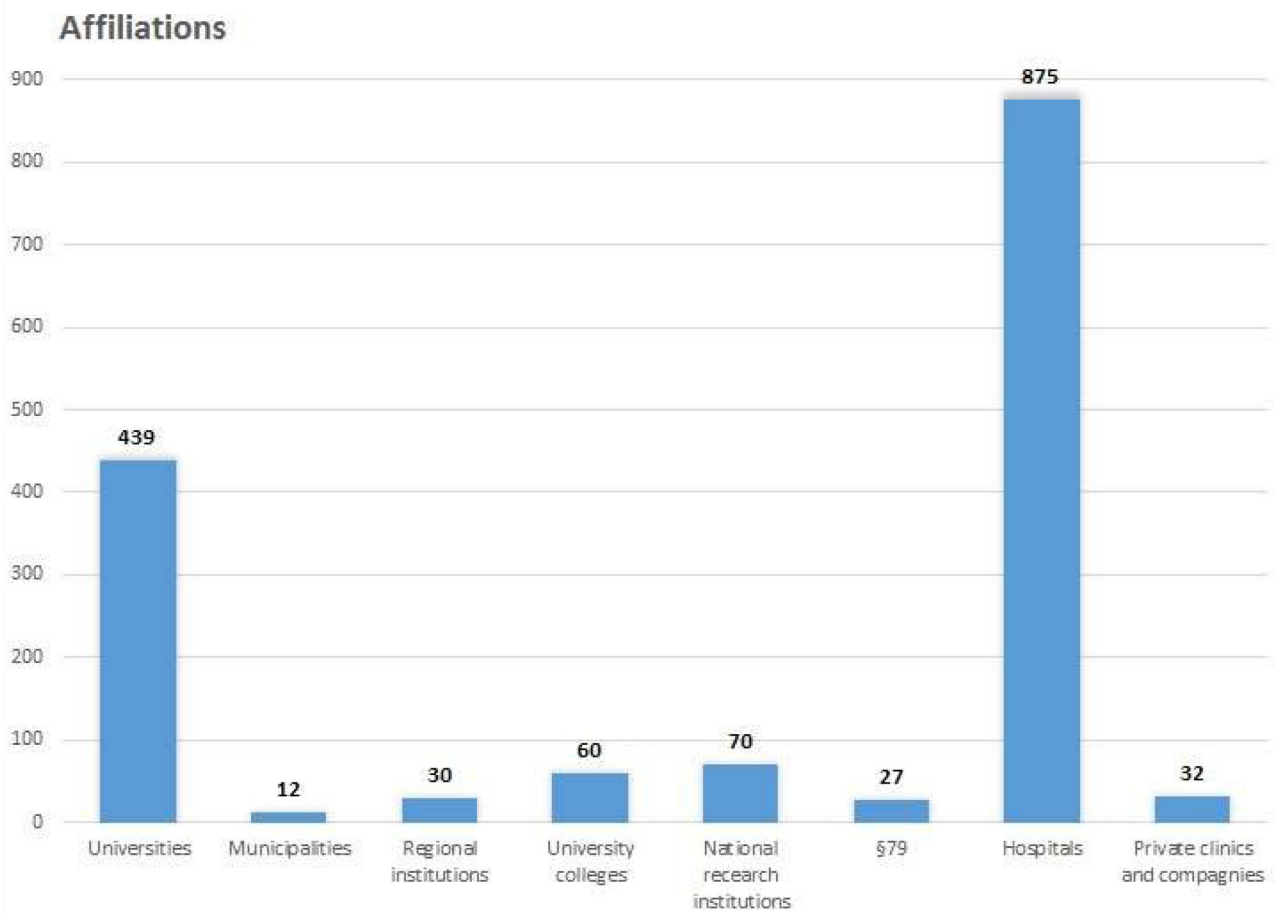
Number of affiliations.

A total of 1,545 (87%) of the included studies had a first author with a primary Danish affiliation. The primary authors providing evidence represented all Danish universities except one (IT University of Copenhagen), public hospitals from all hospital regions, all university colleges, 4 out of 98 municipalities, all relevant national and regional institutions, 15 private clinics, and 5 private companies (Figure 4).

Different institutions contributed a similar amount of research among the groups. However, the qualitative studies exploring HCPs' perspectives were primarily affiliated to universities and hospitals.

Hospitals were the largest contributors to rehabilitation research with a total number of affiliations at 57% of all institutions. All departments and centers at hospitals, such as RH's Neuroscience Center, were categorized as belonging to the hospital.

A further 2% was research affiliated to non-profit and tax-financed specialized institutions, e.g., Multiple Sclerosis hospitals, and The Danish Rheumatism Association's specialized rehabilitation center Sano.

Universities contributed the second highest number of affiliations (28%), followed by national research institutions (5%) (Figure 4). University colleges and private companies contributed 4 and 2%, respectively, usually with one study each. The smallest contributor of affiliations counted was from municipalities at 1%. Among universities, the University of Southern Denmark was the largest affiliation contributor followed by Copenhagen University, Aarhus University, and Aalborg University with 127, 125, 105, and 78, respectively.

Among hospital regions, Capital Region of DK was the largest contributor followed by Central DK Region, Region of Southern DK, Region Zealand, and the North DK Region with 506, 216, 66, 51, and 36, respectively.

Among national institutions, National Research Center for the Working Environment was the largest contributor with 30 affiliations followed by the Danish Cancer Society Research Center with 22 and Danish Knowledge Center for Rehabilitation and Palliative Care with 14.

Among regional research institutions, DEFACTUM, Central DK Region was the largest contributor with 20 publications followed by the Department of Social Medicine, Aarhus with 5.

### Additional Analyses

As the studies with no Danish first author were not counted, a subgroup analysis between the two groups was done to qualify the counted number between study groups. Thus, 20% of 109 studies in the elderly group and only 4 (12%) of 33 studies in the mental/cognitive group have a Danish co-author.

**Figure 5:**
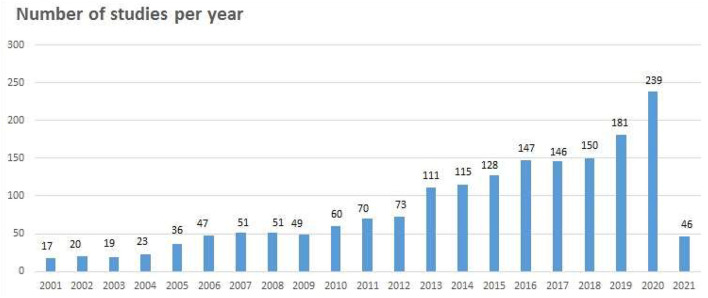
Number of studies per year.

Rehabilitation services and research have developed due to epidemiological, political-administrative, and institutional changes. An analysis of publication year revealed only 17 studies published in 2001, increasing to 239 publications in 2020 (Figure 5). Publication trends show an almost linear development with a 10% yearly increase from 2001 to 2012, rising to 15% per year from 2013 to 2018, and even greater increases after 2019. In the first 2 months of 2021, 46 studies were published.

## Discussion

### Summary of Findings

This scoping review provides the most comprehensive mapping to date of the available published literature on rehabilitation research conducted in Danish contexts gathered over a 20-year period regarding study groups, study designs, and institutions.

A total of 1,779 studies included 24 broad study groups representing types of health conditions and other categories. Rheumatic diseases, diabetes, and substance abuse had a remarkably small number of studies (below 10) considering the prevalence of these groups.

The majority of designs were cohort studies, but all types of study designs were found in most groups. One in ten were secondary studies; however, the number of qualitative secondary studies was sparse.

A majority of studies had a Danish first author (86.8%). The hospitals accounted for almost half of the affiliations, universities contributed to a third, followed by university colleges (4%). In 2007, a large reform in the political and administrative structure led to a displacement in responsibility for providing rehabilitation services from regional hospitals to 98 municipalities. The formal responsibility for research, however, did not follow. That is, most rehabilitation service is performed by the municipalities without research responsibility. The infrastructure of research has changed following an educational reform in 2013. Until then, the universities were the only public research institutions. From 2014 onward, university colleges were approved to undertake research, including rehabilitation research (26).

During the 20-year period, an increase in the number of studies is remarkable, especially from 2013. The university colleges' approval for research in 2014, starting three quarters into the time period studied, may reflect this fact. However, this follows the international publication trend for rehabilitation studies searched by PubMed (27). The increase is expected to continue. From January to February 2021, 46 studies were published; thus, an estimation of 276 studies is to be published in 2021.

### Strengths and Limitations

Regarding the quality, we conducted systematic searches on rehabilitation; therefore, it is unlikely that we missed the important studies that may have substantively changed the conclusions of this overview. Furthermore, processes were standardized for selection and data extraction. However, due to the a number of studies, the interpretation of the findings could have resulted from a different approach to address categories of study groups, e.g., the overlap between the study groups elderly and cardiac, as cardiac-related disease prevalence increases with age (28). Another limitation may be our selection of databases within the fields of healthcare. Scoping reviews do not consider the quality of the included studies.

The total number of studies was an overestimation as several studies had almost the same title. Further, PhD students and other authors were counted with several publications on the same study population/topic/other design.

The criteria stating that “rehab” should be included in the title or abstract may have excluded the studies in the field of rehabilitation, e.g., named prevention, restorative care, or psychosocial intervention (29). Moreover, studies about “everyday rehabilitation” may be missed as the international term “reablement” was introduced by legislation in 2015 (17). There has been no tradition of naming what has been investigated as part of a rehabilitation process, and any specific definition for a purpose will lead to a misclassification (29).

In relation to some neurological diseases e.g., spinal cord injury, the practice in hospitals over the last 30 years has been to merge rehabilitation with treatment from day 1. However, the definitions of rehabilitation during the studied time period may have caused the underestimation of included studies, e.g., among the groups neurological diseases and psychiatry (4, 7). Further, some studies were categorized in “biomedical aspects,” e.g., studies regarding effects on body functions and activities by the modulation of the nervous system by technical or medical means without naming it “rehabilitation.” With significant global demand for rehabilitation, it is crucial that research aimed at functioning actually is called rehabilitation research.

### Categories

The categorization of study groups was inspired by earlier reviews on functioning (30, 31) and specific condition as in a recent scoping review (14). Other classifications, like the rehabilitation research matrix, which provided a less clinical and more sociological approach to rehabilitation research or the review by Cieza et al. could have been used (3, 32). However, the chosen approach made comparisons possible to another scoping review on rehabilitation studies (14), and are similar to those categories in a review regarding the global need of rehabilitation (3).

These reviews also found that studies among persons with MSD were the largest group represented, whereas other authors found a majority of studies not related to a specific health condition (14). The difficulties regarding the categorization of rehabilitation interventions and groups are well-described (8, 33).

The design categories were based on the Cochrane Handbook (25); however, some may have been merged, e.g., surveys and cross-sectional cohort studies. RCTs were included in the secondary group if two RCTs were included.

The method chosen for counting affiliations could have changed the number of Danish institutions involved. The subanalysis revealed that studies with Danish first authorship most often also had a Danish last author.

Misclassification/choice of categories during data extraction may have overestimated and underestimated the number of studies in certain study groups. We found only a small number of studies on diabetes and rheumatic disease. Studies may have been classified in the elderly group or mixed group as the prevalence of both diabetes and rheumatic diseases increases with age (34), and diabetes is also highly associated with multimorbidity (15). Yet, the category elderly included typical diagnoses related to age, such as cardiac disease, dementia, cancer, or hip replacement. More likely, a few studies on rheumatic disease have been classified as the MSD group.

The prevalence of multimorbidity in aging (35) may have increased the number of studies in this category. However, the category was deemed relevant as this demographic factor increases the demand for rehabilitation. Further, the category mixed encompasses elderly persons as multimorbidity increases with age (15). Multimorbidity is prevalent in 20–50% of persons aged 65 and over (15, 35). The UN defines “older persons” as aged 65 and over (36).

Mental/cognitive group studies included persons with psychiatric diagnoses, which could have been a separate group. However, only seven studies (five schizophrenia, one anorexia, and one pervasive refusal syndrome) were included in a group where other studies found a higher proportion (14). Studies among people with dementia could have been categorized in either elderly or neurologic diseases, but there were only six studies.

By consensus and in accordance with the WHO, the relevant outcomes of rehabilitation are functioning and social participation. However, a limitation to this review is that we did not look at the aims and outcomes of the rehabilitation; neither if functioning and participation were measured or whether the outcomes were relevant, e.g., quality of life (QoL) (37). Some will agree that an improved QoL is the ultimate goal although many well-validated health-related QoL instruments are available, they are generally flawed (7). Moreover, QoL may be considered more a defining goal in palliative care than in rehabilitation (38).

Another research question may have mapped to which extent the ICF model is used as a reference. Further, the rehabilitation research map is located primarily at the individual micro level's first or second cell encompassing studies among individuals with disabilities (e.g., life experiences) and knowledge production questioning what works in rehabilitation, respectively (32). Thus, studies regarding the organization of rehabilitation from the third cell are left out (32).

### Recommendation for Research and Practice

Future research should use common categorizes regarding the populations and categories from the global review (3). Among the most common health conditions, there is a need for further well-conducted rehabilitation research, e.g., people living with rheumatic diseases, vulnerable groups, and psychiatric health conditions. Further, the exploration of prevalent public health issues, such as persons living with diabetes and overweight health conditions in the need for rehabilitation, should be conducted.

The study designs illustrate the context; thus, most research was affiliated to hospitals with studies representing a typical hierarchy of design. However, all design types are needed in rehabilitation research, therefore more action research and case studies could be relevant as these include contextual factors and participation outcomes to a higher extent.

Regarding the choice of relevant outcomes, functioning “is the ultimate objective of rehabilitation, regardless of who the beneficiary is, who delivers it, or the context in which rehabilitation is delivered” (3).

The findings lead to a number of relevant issues to explore in future research: Have the aims of rehabilitation changed over time? Which outcomes have been chosen? Is the ICF model used? Which stakeholders are involved? Do studies explore costs in relation to society?

## Conclusion

Following screening, 1,779 studies involving 24 broad study groups were included. The categories were mostly diagnosis-based; MSD, cancer, and cardiac health conditions encompassed most studies. All study designs were represented, 1/10 were secondary studies. The majority (87%) of studies had a Danish first authorship. Affiliations to 119 national, regional, municipal, and private Danish institutions were identified. The majority of affiliations counted were among hospitals followed by universities. A few municipalities were represented although they are not yet approved to conduct research. Publication trends showed an increase in studies primarily from 2013.

## Data Availability Statement

The original contributions presented in the study are included in the article/Supplementary Material, further inquiries can be directed to the corresponding author.

## Author Contributions

A-MHM and JF contributed to review method design, search strategy, study selection, data extraction, data analysis, write-up, and wrote the first draft of this manuscript. TM contributed as a third reviewer, and JT and CN contributed for data extraction and analysis, TM, JT, and CN contributed to a critically revision of the manuscript. All authors contributed to the article and approved the submitted version.

## Funding

This research was supported by the VELUX-FONDEN, jr.nr. 00040238.

## Conflict of Interest

The authors declare that the research was conducted in the absence of any commercial or financial relationships that could be construed as a potential conflict of interest.

## Publisher's Note

All claims expressed in this article are solely those of the authors and do not necessarily represent those of their affiliated organizations, or those of the publisher, the editors and the reviewers. Any product that may be evaluated in this article, or claim that may be made by its manufacturer, is not guaranteed or endorsed by the publisher.

## Supplementary Material

Appendix A. Search history.

Appendix B. PRISMA-scoping review reporting guideline and checklist.

## References

[1] Gimigliano F Negrini S. The world health organization “rehabilitation 2030: a call for action”. Eur J Phys Rehabil Med. (2017) 53:155–68. 10.23736/S1973-9087.17.04746-3 28382807

[2] Stucki G Bickenbach J Gutenbrunner C Melvin J. Rehabilitation: the health strategy of the 21st century. J Rehabil Med. (2018) 50:309–16. 10.2340/16501977-2200 28140419

[3] Cieza A Causey K Kamenov K Hanson SW Chatterji S Vos T. Global estimates of the need for rehabilitation based on the Global Burden of Disease study 2019: a systematic analysis for the Global Burden of Disease Study 2019. Lancet. (2021) 396:2006–17. 10.1016/S0140-6736(20)32340-0 33275908 PMC7811204

[4] Meyer T Kiekens C Selb M Posthumus E Negrini S. Toward a new definition of rehabilitation for research purposes: a comparative analysis of current definitions. Eur J Phys Rehabil Med. (2020) 56:672–81. 10.23736/S1973-9087.20.06610-1 32990687

[5] Stucki G Bickenbach J. 1. 1 Basic concepts, definitions and models. J Int Soc Phys Rehabil Med. (2019) 2:8–12. 10.4103/jisprm.jisprm_5_19

[6] Organisation TWH. International Classification of Functioning, Disability and Health: WHO. (2001). Available online at: https://www.who.int/standards/classifications/international-classification-of-functioning-disability-and-health (accessed February 24, 2022).

[7] Wade DT. What is rehabilitation? an empirical investigation leading to an evidence-based description. Clin Rehabil. (2020) 34:571–83. 10.1177/0269215520905112 32037876 PMC7350200

[8] Negrini S Arienti C Küçükdeveci A Lazzarini SG Patrini M Kiekens C. Current rehabilitation definitions do not allow correct classification of Cochrane systematic reviews: an overview of Cochrane reviews. Eur J Phys Rehabil Med. (2020) 56:667–71. 10.23736/S1973-9087.20.06585-5 32935959

[9] Momsen AH Stapelfeldt CM Rosbjerg R Escorpizo R Labriola M Bjerrum M. International classification of functioning, disability and health in vocational rehabilitation: a scoping review of the state of the field. J Occup Rehabil. (2019) 29:241–73. 10.1007/s10926-018-9788-4 29869054 PMC6531389

[10] Nielsen SS Christensen JR. Occupational therapy for adults with overweight and obesity: mapping interventions involving occupational therapists. Occup Ther Int. (2018) 30:7412686. 10.1155/2018/7412686 30510496 PMC6232807

[11] Nawaz A Skjæret N Helbostad JL Vereijken B Boulton E Svanaes D. Usability and acceptability of balance exergames in older adults: a scoping review. Health Informatics J. (2016) 4 911–31. 10.1177/1460458215598638 26303810

[12] Enemark Larsen A Rasmussen B Christensen JR. Enhancing a client-centred practice with the Canadian occupational performance measure. Occup Ther Int. (2018) 2018:5956301. 10.1155/2018/5956301 30050391 PMC6040242

[13] Tropea P Schlieter H Sterpi I Judica E Gabilondo . Rehabilitation, the great absentee of virtual coaching in medical care: scoping review. J Med Internet Res. (2019) 1:e12805. 10.2196/12805 31573902 PMC6774233

[14] Colquhoun HL Jesus TS O'Brien KK Tricco AC Chui A Zarin W . Scoping review on rehabilitation scoping reviews. Arch Phys Med Rehabil. (2020) 101:1462–9. 10.1016/j.apmr.2020.03.015 32325163

[15] Møller SP Laursen B Johannesen CK Tolstrup JS Schramm S. Patterns of multimorbidity and demographic profile of latent classes in a Danish population-a register-based study. PLoS ONE. (2020) 15:e0237375. 10.1371/journal.pone.0237375 32780781 PMC7418992

[16] Andersen RM Thomsen T Danielsen AK Gögenur I Alkjær T Nordentoft T . Evaluation of abdominal exercises after stoma surgery: a descriptive study. Disabil Rehabil. (2020) 8:1–10. 10.1080/09638288.2020.1771620 [Epub ahead of print]. 32510238

[17] Rostgaard T Graff L. Reablement in. denmark—better help, better quality of life? Innov Aging. (2017) 1:648. 10.1093/geroni/igx004.229930879470

[18] Labour Mo. Reform of early retirement and flexjobs, including the introduction of resource courses, rehabilitation teams, flexi-wage subsidies, etc. (in Danish) (Reform af førtidspension og fleksjob, herunder indførelse af ressourceforløb, rehabiliteringsteam, fleksløntilskud m.v.) (2012). Agreement on a reform of disability pension and flexibility jobs (2012). Available online at: https://star.dk/media/8043/aftale_fop-pdf.pdf (accessed June 30, 2012).

[19] The Danish Health Ministry. Guidance on municipal rehabilitation Vejledning om kommunal rehabilitering]. Available online at: https://www.retsinformation.dk/eli/retsinfo/2011/9439

[20] MarselisborgCentret. The White book on rehabilitation, Rehabilitation in Denmark [Hvidbog om rehabiliteringsbegrebet Rehabilitering i Danmark] Aarhus, Denmark 2004.

[21] Peters MDJ Marnie C Tricco AC Pollock D Munn Z Alexander L„ et al. Updated methodological guidance for the conduct of scoping reviews. JBI Evid Synth. (2020) 10:2119–26. 10.11124/JBIES-20-00167 33038124

[22] Lockwood C Tricco AC. Preparing scoping reviews for publication using methodological guides and reporting standards. Nurs Health Sci. (2020) 22:1–4. 10.1111/nhs.12673 32115893

[23] Tricco AC Lillie E Zarin W O'Brien KK Colquhoun H Levac D . PRISMA extension for scoping reviews (PRISMA-ScR): checklist and explanation. Ann Intern Med. (2018) 169:467–73. 10.7326/M18-0850 30178033

[24] Page MJ McKenzie JE Bossuyt PM Boutron I Hoffmann TC Mulrow CD . The PRISMA 2020 statement: an updated guideline for reporting systematic reviews. BMJ. (2021) 372:n71. 10.1136/bmj.n71 33782057 PMC8005924

[25] Higgins JPT Thomas J Chandler J Cumpston M Li T Page MJ . Cochrane Handbook for Systematic Reviews of Interventions. 2nd ed. Chichester (UK): John Wiley & Sons.

[26] Lindeberg TH Busch H. The University colleges' new role in research development. [Professionshøjskolernes nye rolle inden for forskning og udvikling]. MONA - Matematik- og Naturfagsdidaktik (2016). Available online at: https://tidsskrift.dk/mona/article/view/36391 (accessed February 26, 2022).

[27] PubMed. Available online at: https://pubmed.ncbi.nlm.nih.gov/?term=rehabilitation%5BTitle%2FAbstract%5D&filter=dates.2001%2F1%2F1-2021%2F2%2F28&sort=date&timeline=expanded (accessed February 24, 2022).

[28] Rodgers JL Jones J Bolleddu SI Vanthenapalli S Rodgers LE Shah K . Cardiovascular risks associated with gender and aging. J Cardiovasc Dev Dis. (2019) 6:19. 10.3390/jcdd6020019 31035613 PMC6616540

[29] Wade DT. Defining rehabilitation: an exploration of why it is attempted, and why it will always fail. Clin Rehabil. (2021) 35:1650–6. 10.1177/02692155211028018 34182808 PMC8552408

[30] Jelsma J. Use of the international classification of functioning, disability and health: a literature survey. J Rehabil Med. (2009) 41:1–12. 10.2340/16501977-0300 19197563

[31] Cerniauskaite M Quintas R Boldt C Raggi A Cieza A Bickenbach JE . Systematic literature review on ICF from 2001 to 2009: its use, implementation and operationalisation. Disabil Rehabil. (2011) 33:281–309. 10.3109/09638288.2010.529235 21073361

[32] Solvang PK Hanisch H Reinhardt JD. The rehabilitation research matrix: producing knowledge at micro, meso, and macro levels. Disabil Rehabil. (2017) 39:1983–9. 10.1080/09638288.2016.1212115 27645805

[33] Levack WMM Rathore FA Pollet J Negrini S. One in 11 Cochrane reviews are on rehabilitation interventions, according to pragmatic inclusion criteria developed by Cochrane rehabilitation. Arch Phys Med Rehabil. (2019) 100:1492–8. 10.1016/j.apmr.2019.01.021 30831091

[34] de Thurah A Bremander A Primdahl J. High-quality RMD rehabilitation and telehealth: evidence and clinical practice. Best Pract Res Clin Rheumatol. (2020) 34:101513. 10.1016/j.berh.2020.101513 32307230

[35] Schiøtz ML SA Høst D Glümer C Frølich A. Social disparities in the prevalence of multimorbidity - a register-based population study. BMC Public Health. (2017) 17:422–8. 10.1186/s12889-017-4314-8 28486983 PMC5424300

[36] United Nations DoEaSA Population Division. World Population Ageing 2019: Highlights (2019).

[37] Ravnborg M Storr L. Is “quality of life” a relevant goal in clinical studies of rehabilitation? Ugeskr Laeger. (2008) 170:859–61. 18364174

[38] Stucki G Bickenbach J. Functioning: the third health indicator in the health system and the key indicator for rehabilitation. Eur J Phys Rehabil Med. (2017) 53:134–8. 10.23736/S1973-9087.17.04565-8 28118696

