# Supplementary material for: Rehabilitation Research in Denmark Between 2001 and 2020: A Scoping Review
Source: Front Rehabil Sci. 2022 Mar 10;3:849216. doi: 10.3389/fresc.2022.849216 (PMC9397721; doi:10.3389/fresc.2022.849216)
Supplement: Supplementary file 1 [file Data_Sheet_1.doc]

###### Appendix 1. Search histories for PubMed, PsycINFO, Embase and Cinahl

In total 5.514/5008 ref.

After removal of dublets 3100 references were referred to Covidence (dublets 460)=2640

PubMed

| **Search** | **Query** | **Results** |
| --- | --- | --- |
| #16 | Search: **#11 NOT #12** Filters: **Abstract, Danish, English, from 2001/1/1 - 2021/3/1** | 2,187 |
| #15 | Search: **#11 NOT #12** Filters: **Abstract, Danish, from 2001/1/1 - 2021/3/1** | [115](https://pubmed.ncbi.nlm.nih.gov/?term=%2310+NOT+%2311&filter=simsearch1.fha&filter=dates.2001%2F1%2F1-2021%2F3%2F1&filter=lang.danish&sort=relevance) |
| #15 | Search: **#11 NOT #12** Filters: **Abstract, from 2001/1/1 - 2021/3/1** | 2,196 |
| #14 | Search: **#11 NOT #12** Filters: Abstract | 2,378 |
| #13 | Search: **#11 NOT #12** | 2,440 |
| #12 | Search: **protocol[Title]** | [55,822](https://pubmed.ncbi.nlm.nih.gov/?term=protocol%5BTitle%5D&sort=) |
| #11 | Search: **#4 AND #10** | 2,548 |
| #10 | Search: **#5 OR #6 OR #7 OR #8 OR #9** | 228,734 |
| #9 | Search: **Denmark[Affiliation] OR Danmark[Affiliation] OR DK[Affiliation] OR Danish[Affiliation]** | [221,512](https://pubmed.ncbi.nlm.nih.gov/?term=Denmark%5BAffiliation%5D+OR+Danmark%5BAffiliation%5D+OR+DK%5BAffiliation%5D+OR+Danish%5BAffiliation%5D&sort=) |
| # 8 | Search: **Odense[Affiliation] OR Herlev[Affiliation] OR Bispebjerg[Affiliation] OR Glostrup[Affiliation] OR Esbjerg[Affiliation] OR Herning[Affiliation] OR Viborg[Affiliation] OR Skælskør[Affiliation]** | 48,347 |
| #7 | Search: **"Aalborg"[Affiliation] OR "Ålborg"[Affiliation]** | [13,297](https://pubmed.ncbi.nlm.nih.gov/?term="Aalborg"%5BAffiliation%5D+OR+"Ålborg"%5BAffiliation%5D&sort=relevance) |
| #6 | Search: **"Aarhus"[Affiliation] OR "Århus"[Affiliation]** | [51,917](https://pubmed.ncbi.nlm.nih.gov/?term="Aarhus"%5BAffiliation%5D+OR+"Århus"%5BAffiliation%5D&sort=) |
| #5 | Search: **"Copenhagen"[Affiliation] OR "København"[Affiliation]** | [107,139](https://pubmed.ncbi.nlm.nih.gov/?term="Copenhagen"%5BAffiliation%5D+OR+"København"%5BAffiliation%5D&sort=) |
| #4 | Search: **#1 OR #2 OR #3** | 190,269 |
| #3 | Search: **OR "habilitation"[Title/Abstract] OR "habilita*"[Title/Abstract]** | 1,248 |
| #2 | Search: **"Rehabilitation"[Title/Abstract] OR "rehabilita*"[ Title/Abstract]** | 184,317 |
| #1 | Search: **"Rehabilitation"[MeSH Terms:noexp]** | 18,4323 |

PsycINFO

| **S1** | [MAINSUBJECT.EXACT("Rehabilitation")](https://search-proquest-com.ez.statsbiblioteket.dk:12048/recentsearches.recentsearchtabview.recentsearchesgridview.scrolledrecentsearchlist.checkdbssearchlink:rerunsearch/C590180D6A0466APQ/None?site=psycinfo&t:ac=RecentSearches) | [**21,312**](https://search-proquest-com.ez.statsbiblioteket.dk:12048/recentsearches.recentsearchtabview.recentsearchesgridview.scrolledrecentsearchlist.checkdbssearchlink_0:rerunsearch/C590180D6A0466APQ/None?site=psycinfo&t:ac=RecentSearches) |
| --- | --- | --- |
| **S2** | [ab(Rehabilitation) OR ti(rehabilitation)](https://search-proquest-com.ez.statsbiblioteket.dk:12048/recentsearches.recentsearchtabview.recentsearchesgridview.scrolledrecentsearchlist.checkdbssearchlink:rerunsearch/C855ADA5BBA044CBPQ/None?site=psycinfo&t:ac=RecentSearches) | [**53,984**](https://search-proquest-com.ez.statsbiblioteket.dk:12048/recentsearches.recentsearchtabview.recentsearchesgridview.scrolledrecentsearchlist.checkdbssearchlink_0:rerunsearch/C855ADA5BBA044CBPQ/None?site=psycinfo&t:ac=RecentSearches) |
| **S3** | [ab(Rehabilita*) OR ti(rehabilita*)](https://search-proquest-com.ez.statsbiblioteket.dk:12048/recentsearches.recentsearchtabview.recentsearchesgridview.scrolledrecentsearchlist.checkdbssearchlink:rerunsearch/54E5D3B8BF4E4D04PQ/None?site=psycinfo&t:ac=RecentSearches) | [**57,804**](https://search-proquest-com.ez.statsbiblioteket.dk:12048/recentsearches.recentsearchtabview.recentsearchesgridview.scrolledrecentsearchlist.checkdbssearchlink_0:rerunsearch/54E5D3B8BF4E4D04PQ/None?site=psycinfo&t:ac=RecentSearches) |
| **S4** | [ab(habilita*) OR ti(habilita*)](https://search-proquest-com.ez.statsbiblioteket.dk:12048/recentsearches.recentsearchtabview.recentsearchesgridview.scrolledrecentsearchlist.checkdbssearchlink:rerunsearch/35B4885A448D49B0PQ/None?site=psycinfo&t:ac=RecentSearches) | [**940**](https://search-proquest-com.ez.statsbiblioteket.dk:12048/recentsearches.recentsearchtabview.recentsearchesgridview.scrolledrecentsearchlist.checkdbssearchlink_0:rerunsearch/35B4885A448D49B0PQ/None?site=psycinfo&t:ac=RecentSearches) |
| **S5** | [ab(habilitation) OR ti(habilitation)](https://search-proquest-com.ez.statsbiblioteket.dk:12048/recentsearches.recentsearchtabview.recentsearchesgridview.scrolledrecentsearchlist.checkdbssearchlink:rerunsearch/6C116A9F1A364C2APQ/None?site=psycinfo&t:ac=RecentSearches) | [**747**](https://search-proquest-com.ez.statsbiblioteket.dk:12048/recentsearches.recentsearchtabview.recentsearchesgridview.scrolledrecentsearchlist.checkdbssearchlink_0:rerunsearch/6C116A9F1A364C2APQ/None?site=psycinfo&t:ac=RecentSearches) |
| **S6** | [MAINSUBJECT.EXACT("Rehabilitation") OR (ab(Rehabilitation) OR ti(rehabilitation)) OR (ab(Rehabilita*) OR ti(rehabilita*)) OR (ab(habilita*) OR ti(habilita*)) OR (ab(habilitation) OR ti(habilitation))](https://search-proquest-com.ez.statsbiblioteket.dk:12048/recentsearches.recentsearchtabview.recentsearchesgridview.scrolledrecentsearchlist.checkdbssearchlink:rerunsearch/2C539B877A7B4F4BPQ/None?site=psycinfo&t:ac=RecentSearches) | [**62,206**](https://search-proquest-com.ez.statsbiblioteket.dk:12048/recentsearches.recentsearchtabview.recentsearchesgridview.scrolledrecentsearchlist.checkdbssearchlink_0:rerunsearch/2C539B877A7B4F4BPQ/None?site=psycinfo&t:ac=RecentSearches) |
| **S7** | [af(Denmark) OR af(Danmark) OR af(DK) OR af(Danish)](https://search-proquest-com.ez.statsbiblioteket.dk:12048/recentsearches.recentsearchtabview.recentsearchesgridview.scrolledrecentsearchlist.checkdbssearchlink:rerunsearch/AEB48AF99C7C43E7PQ/None?site=psycinfo&t:ac=RecentSearches) | [**19,893**](https://search-proquest-com.ez.statsbiblioteket.dk:12048/recentsearches.recentsearchtabview.recentsearchesgridview.scrolledrecentsearchlist.checkdbssearchlink_0:rerunsearch/AEB48AF99C7C43E7PQ/None?site=psycinfo&t:ac=RecentSearches) |
| **S8** | [af(Copenhagen) OR af(København)](https://search-proquest-com.ez.statsbiblioteket.dk:12048/recentsearches.recentsearchtabview.recentsearchesgridview.scrolledrecentsearchlist.checkdbssearchlink:rerunsearch/EED690E8F44F49DEPQ/None?site=psycinfo&t:ac=RecentSearches) | [**11,561**](https://search-proquest-com.ez.statsbiblioteket.dk:12048/recentsearches.recentsearchtabview.recentsearchesgridview.scrolledrecentsearchlist.checkdbssearchlink_0:rerunsearch/EED690E8F44F49DEPQ/None?site=psycinfo&t:ac=RecentSearches) |
| **S9** | [af(Aarhus) OR af(Århus)](https://search-proquest-com.ez.statsbiblioteket.dk:12048/recentsearches.recentsearchtabview.recentsearchesgridview.scrolledrecentsearchlist.checkdbssearchlink:rerunsearch/665BBC75CE534688PQ/None?site=psycinfo&t:ac=RecentSearches) | [**7,833**](https://search-proquest-com.ez.statsbiblioteket.dk:12048/recentsearches.recentsearchtabview.recentsearchesgridview.scrolledrecentsearchlist.checkdbssearchlink_0:rerunsearch/665BBC75CE534688PQ/None?site=psycinfo&t:ac=RecentSearches) |
| **S10** | [af(Aalborg) OR af(Ålborg)](https://search-proquest-com.ez.statsbiblioteket.dk:12048/recentsearches.recentsearchtabview.recentsearchesgridview.scrolledrecentsearchlist.checkdbssearchlink:rerunsearch/E9499175E3294F90PQ/None?site=psycinfo&t:ac=RecentSearches) | [**2,583**](https://search-proquest-com.ez.statsbiblioteket.dk:12048/recentsearches.recentsearchtabview.recentsearchesgridview.scrolledrecentsearchlist.checkdbssearchlink_0:rerunsearch/E9499175E3294F90PQ/None?site=psycinfo&t:ac=RecentSearches) |
| **S11** | [af(Odense) OR af(Herlev) OR af(Bispebjeg) OR af(Glostrup) OR af(Esbjerg) OR af(Herning) OR af(Viborg) OR af(Skælskør)](https://search-proquest-com.ez.statsbiblioteket.dk:12048/recentsearches.recentsearchtabview.recentsearchesgridview.scrolledrecentsearchlist.checkdbssearchlink:rerunsearch/2F8C70EDC63B4366PQ/None?site=psycinfo&t:ac=RecentSearches) | [**2,298**](https://search-proquest-com.ez.statsbiblioteket.dk:12048/recentsearches.recentsearchtabview.recentsearchesgridview.scrolledrecentsearchlist.checkdbssearchlink_0:rerunsearch/2F8C70EDC63B4366PQ/None?site=psycinfo&t:ac=RecentSearches) |
| **S12** | [(af(Denmark) OR af(Danmark) OR af(DK) OR af(Danish)) OR (af(Copenhagen) OR af(København)) OR (af(Aarhus) OR af(Århus)) OR (af(Aalborg) OR af(Ålborg)) OR (af(Odense) OR af(Herlev) OR af(Bispebjeg) OR af(Glostrup) OR af(Esbjerg) OR af(Herning) OR af(Viborg) OR af(Skælskør))](https://search-proquest-com.ez.statsbiblioteket.dk:12048/recentsearches.recentsearchtabview.recentsearchesgridview.scrolledrecentsearchlist.checkdbssearchlink:rerunsearch/FE365881426E4013PQ/None?site=psycinfo&t:ac=RecentSearches) | [**27,320**](https://search-proquest-com.ez.statsbiblioteket.dk:12048/recentsearches.recentsearchtabview.recentsearchesgridview.scrolledrecentsearchlist.checkdbssearchlink_0:rerunsearch/FE365881426E4013PQ/None?site=psycinfo&t:ac=RecentSearches) |
| **S13** | [(MAINSUBJECT.EXACT("Rehabilitation") OR (ab(Rehabilitation) OR ti(rehabilitation)) OR (ab(Rehabilita*) OR ti(rehabilita*)) OR (ab(habilita*) OR ti(habilita*)) OR (ab(habilitation) OR ti(habilitation))) AND ((af(Denmark) OR af(Danmark) OR af(DK) OR af(Danish)) OR (af(Copenhagen) OR af(København)) OR (af(Aarhus) OR af(Århus)) OR (af(Aalborg) OR af(Ålborg)) OR (af(Odense) OR af(Herlev) OR af(Bispebjeg) OR af(Glostrup) OR af(Esbjerg) OR af(Herning) OR af(Viborg) OR af(Skælskør)))](https://search-proquest-com.ez.statsbiblioteket.dk:12048/recentsearches.recentsearchtabview.recentsearchesgridview.scrolledrecentsearchlist.checkdbssearchlink:rerunsearch/54D70EC9951E43A5PQ/None?site=psycinfo&t:ac=RecentSearches) | [**588**](https://search-proquest-com.ez.statsbiblioteket.dk:12048/recentsearches.recentsearchtabview.recentsearchesgridview.scrolledrecentsearchlist.checkdbssearchlink_0:rerunsearch/54D70EC9951E43A5PQ/None?site=psycinfo&t:ac=RecentSearches) |
| **S14** | [(MAINSUBJECT.EXACT("Rehabilitation") OR (ab(Rehabilitation) OR ti(rehabilitation)) OR (ab(Rehabilita*) OR ti(rehabilita*)) OR (ab(habilita*) OR ti(habilita*)) OR (ab(habilitation) OR ti(habilitation))) AND ((af(Denmark) OR af(Danmark) OR af(DK) OR af(Danish)) OR (af(Copenhagen) OR af(København)) OR (af(Aarhus) OR af(Århus)) OR (af(Aalborg) OR af(Ålborg)) OR (af(Odense) OR af(Herlev) OR af(Bispebjeg) OR af(Glostrup) OR af(Esbjerg) OR af(Herning) OR af(Viborg) OR af(Skælskør)))](https://search-proquest-com.ez.statsbiblioteket.dk:12048/recentsearches.recentsearchtabview.recentsearchesgridview.scrolledrecentsearchlist.checkdbssearchlink:rerunsearch/F33E237300C34E8CPQ/None?site=psycinfo&t:ac=RecentSearches)Limits applied | [**566**](https://search-proquest-com.ez.statsbiblioteket.dk:12048/recentsearches.recentsearchtabview.recentsearchesgridview.scrolledrecentsearchlist.checkdbssearchlink_0:rerunsearch/F33E237300C34E8CPQ/None?site=psycinfo&t:ac=RecentSearches) |
| **S15** | [(MAINSUBJECT.EXACT("Rehabilitation") OR (ab(Rehabilitation) OR ti(rehabilitation)) OR (ab(Rehabilita*) OR ti(rehabilita*)) OR (ab(habilita*) OR ti(habilita*)) OR (ab(habilitation) OR ti(habilitation))) AND ((af(Denmark) OR af(Danmark) OR af(DK) OR af(Danish)) OR (af(Copenhagen) OR af(København)) OR (af(Aarhus) OR af(Århus)) OR (af(Aalborg) OR af(Ålborg)) OR (af(Odense) OR af(Herlev) OR af(Bispebjeg) OR af(Glostrup) OR af(Esbjerg) OR af(Herning) OR af(Viborg) OR af(Skælskør)))](https://search-proquest-com.ez.statsbiblioteket.dk:12048/recentsearches.recentsearchtabview.recentsearchesgridview.scrolledrecentsearchlist.checkdbssearchlink:rerunsearch/6BF349FC29F046EEPQ/None?site=psycinfo&t:ac=RecentSearches)Limits applied | [**527**](https://search-proquest-com.ez.statsbiblioteket.dk:12048/recentsearches.recentsearchtabview.recentsearchesgridview.scrolledrecentsearchlist.checkdbssearchlink_0:rerunsearch/6BF349FC29F046EEPQ/None?site=psycinfo&t:ac=RecentSearches) |

###### Embase

|  |  | **Results** |
| --- | --- | --- |
| #13 | ((('rehabilitation'/de OR 'rehabilitation':ab,ti OR ('rehabilita*':ab,ti OR 'habilitation':ab,ti OR 'habilita*':ab,ti)) AND (denmark:ff OR danmark:ff OR dk:ff OR danish:ff OR copenhagen:ff OR københavn:ff OR aarhus:ff OR århus:ff OR aalborg:ff OR ålborg:ff OR odense:ff OR herlev:ff OR bispebjeg:ff OR glostrup:ff OR esbjerg:ff OR herning:ff OR viborg:ff OR skælskør:ff)) AND ('article'/it OR 'article in press'/it OR 'review'/it)) NOT protocol AND [2001-2021]/py AND ([danish]/lim OR [english]/lim) AND [abstracts]/lim | 1756 |
| #12 | ((('rehabilitation'/de OR 'rehabilitation':ab,ti OR ('rehabilita*':ab,ti OR 'habilitation':ab,ti OR 'habilita*':ab,ti)) AND (denmark:ff OR danmark:ff OR dk:ff OR danish:ff OR copenhagen:ff OR københavn:ff OR aarhus:ff OR århus:ff OR aalborg:ff OR ålborg:ff OR odense:ff OR herlev:ff OR bispebjeg:ff OR glostrup:ff OR esbjerg:ff OR herning:ff OR viborg:ff OR skælskør:ff)) AND ('article'/it OR 'article in press'/it OR 'review'/it)) NOT protocol AND [2001-2021]/py AND ([danish]/lim OR [english]/lim) | 1769 |
| #11 | ((('rehabilitation'/de OR 'rehabilitation':ab,ti OR ('rehabilita*':ab,ti OR 'habilitation':ab,ti OR 'habilita*':ab,ti)) AND (denmark:ff OR danmark:ff OR dk:ff OR danish:ff OR copenhagen:ff OR københavn:ff OR aarhus:ff OR århus:ff OR aalborg:ff OR ålborg:ff OR odense:ff OR herlev:ff OR bispebjeg:ff OR glostrup:ff OR esbjerg:ff OR herning:ff OR viborg:ff OR skælskør:ff)) AND ('article'/it OR 'article in press'/it OR 'review'/it)) NOT protocol AND [2001-2021]/py | 1772 |
| #10 | ((('rehabilitation'/de OR 'rehabilitation':ab,ti OR ('rehabilita*':ab,ti OR 'habilitation':ab,ti OR 'habilita*':ab,ti)) AND (denmark:ff OR danmark:ff OR dk:ff OR danish:ff OR copenhagen:ff OR københavn:ff OR aarhus:ff OR århus:ff OR aalborg:ff OR ålborg:ff OR odense:ff OR herlev:ff OR bispebjeg:ff OR glostrup:ff OR esbjerg:ff OR herning:ff OR viborg:ff OR skælskør:ff)) AND ('article'/it OR 'article in press'/it OR 'review'/it)) NOT protocol | 1942 |
| #9 | Protocol | 585601 |
| #8 | (('rehabilitation'/de OR 'rehabilitation':ab,ti OR ('rehabilita*':ab,ti OR 'habilitation':ab,ti OR 'habilita*':ab,ti)) AND (denmark:ff OR danmark:ff OR dk:ff OR danish:ff OR copenhagen:ff OR københavn:ff OR aarhus:ff OR århus:ff OR aalborg:ff OR ålborg:ff OR odense:ff OR herlev:ff OR bispebjeg:ff OR glostrup:ff OR esbjerg:ff OR herning:ff OR viborg:ff OR skælskør:ff)) AND ('article'/it OR 'article in press'/it OR 'review'/it) | 2101 |
| #7 | ('rehabilitation'/de OR 'rehabilitation':ab,ti OR ('rehabilita*':ab,ti OR 'habilitation':ab,ti OR 'habilita*':ab,ti)) AND (denmark:ff OR danmark:ff OR dk:ff OR danish:ff OR copenhagen:ff OR københavn:ff OR aarhus:ff OR århus:ff OR aalborg:ff OR ålborg:ff OR odense:ff OR herlev:ff OR bispebjeg:ff OR glostrup:ff OR esbjerg:ff OR herning:ff OR viborg:ff OR skælskør:ff) | 2873 |
| #6 | denmark:ff OR danmark:ff OR dk:ff OR danish:ff OR copenhagen:ff OR københavn:ff OR aarhus:ff OR århus:ff OR aalborg:ff OR ålborg:ff OR odense:ff OR herlev:ff OR bispebjeg:ff OR glostrup:ff OR esbjerg:ff OR herning:ff OR viborg:ff OR skælskør:ff | 292787 |
| #5 | denmark OR danmark OR dk OR danish OR copenhagen OR københavn OR aarhus OR århus OR aalborg OR ålborg OR odense OR herlev OR bispebjeg OR glostrup OR esbjerg OR herning OR viborg OR skælskør | 639682 |
| #4 | 'rehabilitation'/de OR 'rehabilitation':ab,ti OR ('rehabilita*':ab,ti OR 'habilitation':ab,ti OR 'habilita*':ab,ti) | 280806 |
| #3 | 'rehabilita*':ab,ti OR 'habilitation':ab,ti OR 'habilita*':ab,ti | 255576 |
| #2 | 'rehabilitation':ab,ti | 241777 |
| #1 | 'rehabilitation'/de | 91311 |

CIHANL

S7 S3 AND S4 Limiters - Abstract Available; Published Date: 20010101-20211231;

Language: Danish, English.

Search modes - Boolean/Phrase; Interface - EBSCOhost Research

- Advanced Search Database - CINAHL with Full Text **1,044**
